# Effect of Cigarette and Shisha smoking on cognitive functions impairment: A cross sectional study

**DOI:** 10.12669/pjms.36.5.2251

**Published:** 2020

**Authors:** Shahid Bashir, Ghulam Murtaza, Sultan Ayoub Meo, Abeer Al-Masri

**Affiliations:** 1 Shahid Bashir Berenson-Allen Centre for non Invasive brain stimulation, Boston, MA, Harvard Medical School, USA; 2 Ghulam MurtazaDepartment of Zoology, University of Gujrat, Gujrat, Pakistan; 3 Sultan Ayoub Meo, Department of Physiology, College of Medicine, King Saud University, Riyadh, Saudi Arabia; 4 Abeer Al-Masri, Department of Physiology, College of Medicine, King Saud University, Riyadh, Saudi Arabia

**Keywords:** Cognitive functions, Shisha, Cigarette, Smoking, CANTAB

## Abstract

**Objectives:**

Cigarette and Shisha smoking is becoming a common practice in young generation worldwide. Since, this is a growing threat to public health, our study aims to investigate the cognitive function responses of cigarette and Shisha inhalation in adolescents.

**Methods:**

This retrospective cross sectional study comprised three groups, cigarette smoker, Shisha smoker, and nonsmoker control group (each n=25). All the participants were apparently healthy male volunteers aged 21-24 years. Cognitive functions were assessed by employing “Cambridge Neuropsychological Test Automated Battery”. The cognitive functions outcome variables were response time tasks (attention switching task (AST) and the percentage of correct answers pattern recognition memory (PRM) task.

**Results:**

Cigarette and Shisha smokers exhibited a considerable decline in cognitive performance parameters, AST mean correct latency (*p*=0.001), AST mean correct latency (congruent) (*p*=0.001), AST mean correct latency (incongruent) (*p*=0.001) and AST mean correct latency (switching) (*p*=0.001) compared to matched control group.

**Conclusions:**

Cigarette and Shisha smokers exhibited significant impairment in their cognitive functions. The present study findings convince that cigarette and Shisha smokers should quit smoking.

## INTRODUCTION

Cigarette and Shisha smoking is frequently used among young adolescence.^1^ The use of Shisha smoking is about 5%-17% and 6%-34% in American and Middle-Eastern adolescents, respectively. The studies have reported that its prevalence has reached up to 44.3% in the Middle Eastern countries.^2^ In Shisha smoke, large amount of fruit-flavored substances such as “coconut, plum, apple, mango, mint, cola, and strawberry and nicotine, carbon monoxide, fine and ultrafine particulate matter, volatile aldehydes, carcinogenic polycyclic aromatic hydrocarbons, and phenolic compounds are present”. Moreover, heavy metals such as lead and arsenic are also present in Shisha smoke^3^-^4^ which are frequently associated with several diseases.^5^ Cigarette contains 1%-3% nicotine while Shisha tobacco has about 2%-4%. Moreover, 0.34%-1.40% carbon monoxide is present in Shisha smoke.^6^ Presently, electronic cigarettes are also swiftly becoming an alternative form of cigarette smoking worldwide.^7^ Tobacco has been linked with many illnesses, carcinomas^8^ and high mortality.^9^

The trend of using cigarette and Shisha smoking is rapidly increasing in university adolescence worldwide. Therefore, in this study, computerized standard software, “Cambridge Neuropsychological Test Automated Battery (CANTAB)”, was employed to investigate the cognitive functions. Its application has an advantage, as it is a computerized test and takes less time doing the task. Moreover, it gives more accurate results especially in tasks requiring counting time and response delay like attention switching task (AST) compared to traditional pen-and-paper cognitive assessment tasks. “AST and pattern recognition memory (PRM) tests were conducted to investigate the cognitive functions as the “memory and attention” are particularly of the main parameters in assessing cognition in smokers.^10^ Based on modulatory impact of cigarette and Shisha smoking on cognition, we hypothesized that cigarette and Shisha smoking would be linked with decrease in cognitive performance among university adolescents.

## METHODS

This retrospective quasi experimental cross-sectional study was carried out in the “College of Medicine, King Saud University, Riyadh, Saudi Arabia” during November 2015-May 2016. Age, gender, ethnicity, and socioeconomic status-matched design was employed to assess the association between Shisha and cigarette smoking on cognitive functions.

### Selection of Subjects

After getting ethical approval, the investigator’s team visited the various Shisha smoking cafes and got permission from the owner to interview the Shisha smokers. Selection of participants was based on their volunteer participation. Subjects for this study were enrolled after a detailed interview. Similarly, the cigarette group was selected however; the control group was selected from the university students and research employees.

### Exclusion Criteria

All the subjects were asked about their different habits such as Shisha and cigarette smoking, consumption of other tobacco products. Subjects with identified cases of diabetes mellitus, marked obesity, anemia, obstructive lung diseases, malignancy, and chronic alcoholics were excluded. Subjects with known cases of nervousness, difficulty in vision, attention, psychiatric problems, seizures, musculo-skeletal disorders and those on sedatives, hypnotics and who had disturbed sleep history, were excluded from the study.^1^,^7^ We had three groups (n=25 in each group) namely cigarette smoker, Shisha smoker, and control (nonsmoker) group. All were male participants with mean age of 23.12±3.82 (mean±standard deviation) years for cigarette smoker group; 24.7±2.50 years for Shisha smoker, and 22.9±3.43 years for nonsmokers (control) group. All groups were matched for age, ethnicity, gender, and socioeconomic status. In control group, university employees and students were included.

### Cognitive function

“CANTAB research suite software, version 6.0.37, Cambridge cognition” was employed to carry out neuropsychological testing. All the tests took 25-30 minutes for completion. The participants sat comfortably on a seat during testing. They were asked to use the index finger of their dominant hand to press the response button.

### Attention switching task

This test demonstrates the ability of the participants to switch attention between the position and direction of the arrow. In this test, an arrow was displayed on the screen. The participants were asked to indicate the direction and position of the arrow. This test is used to assess the cognitive functions, which is controlled by prefrontal cortex part of the brain and determines the executive dysfunctioning. Congruent and incongruent trials were performed. In congruent trials, the arrow appeared on the right side of the screen, which pointed to the right. In incongruent trials, the arrow appeared on the right side of the screen but pointed towards the left.

### Pattern recognition memory

This test is applied to assess the memory of the participants. In this test, the different visual patterns were displayed on the center of the monitor. The subjects were asked to select one specific pattern among the two patterns displayed on the monitor.

### Ethical Approval

The study was approved by the “Review Committee, College of Medicine, King Saud University, Riyadh, Saudi Arabia (CMED-305-MB12-2016-17)”, and written consent was obtained from the participants.

### Statistical Analysis

SPSS software (version 22.0; SPSS Inc., Chicago, Illinois, USA) was used to perform the statistical analysis. Numerical data was expressed as mean, and standard deviation (SD). We used one-way analysis of variance (ANOVA) to compare data from three groups (cigarette smokers, Shisha smokers and nonsmokers). The *post-hoc* multiple comparison procedures were performed with Tukey’s HSD between the groups. The *p*values less than 0.05 were considered to be statistically significant

## RESULTS

**Fig.1:**
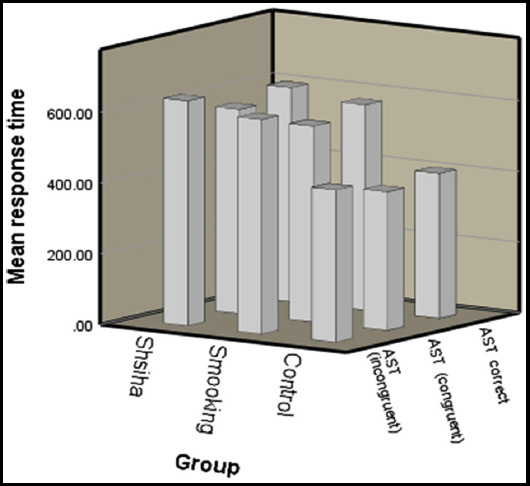
The mean response time (ms) values of AST mean correct latency, AST mean correct latency (congruent) and AST mean correct latency (incongruent) condition for three groups (Shisha smoker, cigarette smoker and control). The error bar showed standard deviation (SD).

The demographic description of the participants is shown in Table-I. In this study, all the participants were males. The values of “AST mean correct latency, AST mean correct latency (congruent) and AST mean correct latency (incongruent)” were recorded as shown in Fig.1.

**Table-I:** Demographic Characteristics.

Group	Numbers	Age (Years) (Mean±SD)
Shisha smoker	25	24.72±2.50
Cigarette smoker	25	23.12±3.82
Nonsmoker	25	22.96±3.43

SD: Standard deviation.

The ANOVA revealed a significant main effect for AST congruency cost (mean correct) (F = 3.406; *p*=0.039), AST mean correct latency (F = 32.859; *p*=0.000), AST mean correct latency (congruent) (F = 33.211; *p*=0.000), AST mean correct latency (incongruent) (F = 29.067; *p*=0.000), AST mean correct latency (blocks 3, 5) (non-switching blocks) (F = 11.641; *p*=0.000) and AST mean correct latency (block 7) (switching block) (F = 41.163; *p*=0.000) (Table-II, Figure 1). There was no significant effect among groups for AST switching cost (F = 3.107; *p*=0.051), AST percent correct trials (F = .554; *p*=0.577) and PRM percent correct (F = 1.773; *p*=0.177, Table-II).

**Table-II:** Cognitive function assessment among control, Shisha and cigarette smokers.

Cognitive Functions Parameters	Mean±SD				

	Controls	Shisha smokers	Cigarette smokers	F-value	p-value
AST congruency cost (mean, correct)	86±56	61±38	55±36	3.406	0.039
AST switching cost (mean, correct)	304±122	297±118	230±103	3.107	0.051
AST mean correct latency	410±61	605±93	580±114	32.859	0.000
AST mean correct latency (congruent)	391±53	577±87	553±112	33.211	0.000
AST mean correct latency (incongruent)	430±75	635±105	606±121	29.067	0.000
AST mean correct latency (blocks 3,5) (non switching blocks)	369±55	461±77	465±100	11.641	0.000
AST mean correct latency (block 7) (switching block)	450±77	759±140	696±151	41.163	0.000
AST percent correct trials	95±4	94 3	94±5	.554	0.577
PRM percent correct	81±7	87±9	88±7	1.773	0.177

AST mean correct latency (p=0.001), AST mean correct latency (congruent) (p=0.001), AST mean correct latency (incongruent) (p=0.001) and AST mean correct latency (switching) (p=0.001) compared to matched control group.

In Shisha smoker versus control group comparison, there was significant difference for the AST mean correct latency (*p*=0.000), AST mean correct latency (congruent) (*p*=0.000), AST mean correct latency (incongruent) (*p*=0.000), AST mean correct latency (blocks 3, 5) (non-switching blocks) (*p*=0.000) and AST mean correct latency (block 7) (switching block) (*p*=0.000) (Table-III). There was not significant effect between two groups for AST congruency cost (*p*=0.130), AST switching cost (*p*=0.980), AST percent correct trials (*p*=0.641) and PRM percent correct (*p*=0.198, Table-III).

**Table-III:** Comparison of various cognitive function test parameters between Shisha, cigarette smokers compared to their matched control subjects.

Cognitive Functions Parameters	P (LSD)			

	Shisha smokers versus control 1 versus 2	Cigarette smokers versus control 2 versus 3	Shisha smokers versus Cigarette smokers 1 versus 3
AST congruency cost (mean, correct)	0.130	0.042	0.880
AST switching cost (mean, correct)	0.980	0.070	0.106
AST mean correct latency	0.000	0.000	0.605
AST mean correct latency (congruent)	0.000	0.000	0.605
AST mean correct latency (incongruent)	0.000	0.000	0.578
AST mean correct latency (blocks 3, 5) (non-switching blocks)	0.000	0.000	0.983
AST mean correct latency (block 7) (switching block)	0.000	0.000	0.193
AST percent correct trials	0.641	0.629	1.000
PRM percent correct	0.198	0.302	0.971

AST: Attention switching task, PRM: Pattern recognition memory, LSD: Least significant difference.

In Cigarette smoker versus control group comparison, there was significant difference for AST congruency cost (*p*=0.042), AST mean correct latency (*p*=0.000), AST mean correct latency (congruent) (*p*=0.000), AST mean correct latency (incongruent) (*p*=0.000), AST mean correct latency (blocks 3, 5) (non-switching blocks) (*p*=0.000) and AST mean correct latency (block 7) (switching block) (*p*=0.000) (Table-III). There was no significant effect among two group for AST switching cost (*p*=0.070), AST percent correct trials (*p*=0.629) and PRM percent correct (*p*=0.302, Table-III).

In Shisha smokers versus cigarette smoker groups, values of AST mean correct latency (congruent), AST mean correct latency (incongruent), AST congruency cost, AST mean correct latency, and AST mean correct latency (block 7) (switching block) were higher in Shisha smokers but not significantly different among group. In other tests, values of AST mean correct latency (blocks 3, 5) (non-switching blocks), AST percent correct trials, and PRM percent correct were higher in cigarette smokers but not significantly different Table-III.

## DISCUSSION

Cigarette and Shisha smoking is becoming a common practice in young generation. It severely affects indoor and outdoor air quality and has adverse impact on the human health. In this study, we investigated the impact of Shisha and cigarette smoking on cognitive performance among adolescents and findings from both groups were compared with the results from control group (nonsmokers). It was identified that Shisha and cigarette smoking reduced the cognitive functions compared to nonsmokers. The present study findings reveal that use of Shisha smoking adversely affects attention, speed, and response time. This may be attributed to dysfunctioning of executive functions, which is controlled by frontal lobe of the brain.^1^

Meo et al.^1^ conducted a study on age, education, income, and gender-matched subjects and reported that adult Shisha smokers had significant impairment in their cognitive functions compared to the matched control group. In previous studies, impact of cigarette smoking on cognitive skills has been investigated. For instance, Doiron et al.^11^ demonstrated that cigarette smoking was allied with cognitive impairment. Jacobsen and colleagues et al.^12^ examined the link between cigarette smoking status and various domains of cognitive function in elderly community-dwelling subjects. The cognitive function impairment was demonstrated even in those subjects who had quit cigarette smoking.

Similarly, Fried et al.^13^ conducted a study on age, education, income, and gender-matched subjects and found that cigarette users exhibited significant cognitive impairments on different tested parameters, which included spatial working memory, and executive planning. Glass et al.^14^ demonstrated that compared to nonsmokers, middle-aged male smokers had faster cognitive decline in global cognition and executive function. Timothy et al.^15^ described that cigarette smoking was related to deficiencies in cognitive flexibility, executive functions, working memory, general intellectual abilities, learning and memory processing speed. In line with previous studies, we observed that cigarette and Shisha smoking significantly affected neurocognitive functions.

In cigarette versus control group, there was no significant difference in PRM, which was in accordance with a previous study by Bashir et al.^16^ This can be attributed to preservation of memory functions in cigarette smokers. In line with our results, cognitive problems due to cigarette smoking in adults have also been demonstrated in various studies.^7^,^11^,^12^ Cigarette smokers showed marked impairment in cognition in sustained attention, spatial working memory, inappropriate behavior, and executive planning domains in comparison with matched nonsmoker group in terms of age, income, gender, and education.^11^ However, we have employed well-validated translational cognitive tasks for evaluation of effects of Shisha and cigarette smoking on cognitive functions, which distinguishes our work from previous studies. The different forms of smoking are rapidly spreading globally, gaining popularity in the Middle East region particularly. With respect to Shisha and cigarette smoking-related investigation on cognitive functions, this work is being reported for the first time in the Middle East region.

**Fig.2:**
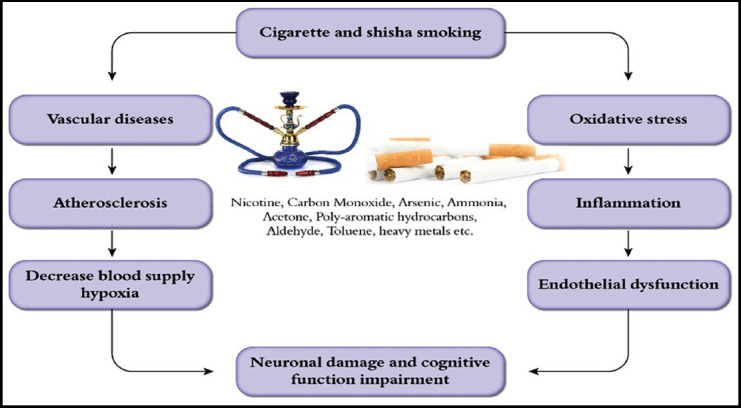
Mechanism how Shisha and cigarette smoking can impair cognitive functions.

The most probable mechanism in line with cigarette, Shisha smoking, and cognitive impairment is the presence of potentially cytotoxic substances which include aldehydes, ketones, carbon monoxide, nitrosamines, dihydroxy benzenes.^17^ These compunds induce oxidation damage to injure the glial or neuronal cells.^16^,^18^ These cytotoxic compounds may affect the neuronal and cellular membrane function of cerebral hemisphere. It has also been demonstrated that smoking evokes cell injury and causes brain function loss with significant effects in the hippocampus.^18^ In line with the above-mentioned studies, we believe that these are the basic factors behind impairment of cognitive functions due to cigarette and Shisha smoking (Fig.2).

### Study strengths and limitations

This work has some strengths and limitations. The major strengths of this study are that, to our best familiarity, no study exists yet, which describes the link between the Shisha and cigarette smoking with cognitive function impairment. The literature is available exploring the neurocognitive consequences of cigarette smoking in middle aged people, there is a specific shortage of studies in the young adults. This is the first study added in the literature on Shisha, cigarette smoking and cognitive functions. The limitation of the present study is the involvement of limited small sample size of Shisha, and cigarette smokers because most of the adolescents were using both Shisha and cigarette, and due to cross-sectional design we could not establish the causation.

## CONCLUSIONS

The young adult cigarette and Shisha smokers exhibited significant impairment in their cognitive functions compared to their matched control group. The present study findings would convince that both cigarette and Shisha smokers should quit smoking. Electronic and print media should also play their role in conveying the message that cigarette and Shisha smoking should not be a part of cultural identity, and avoid social acceptability and perception that Shisha is less harmful or less addictive than cigarettes.

### Authors’ Contributions

**SB:** Study design, supervised the project, data analysis and manuscript writing.

**GM, AAM:** Literature review, subject selections.

**SAM:** Technical support and manuscript writing. All authors have approved the final version of the manuscript.
